# Microglia and Macrophages in Neuroprotection, Neurogenesis, and Emerging Therapies for Stroke

**DOI:** 10.3390/cells10123555

**Published:** 2021-12-16

**Authors:** Susanna R. Var, Anala V. Shetty, Andrew W. Grande, Walter C. Low, Maxim C. Cheeran

**Affiliations:** 1 Department of Neurosurgery, University of Minnesota Medical School, Minneapolis, MN 55455, USA; svar@umn.edu (S.R.V.); grande@umn.edu (A.W.G.); 2 Department of Veterinary Population Medicine, University of Minnesota, St. Paul, MN 55108, USA; 3 Stem Cell Institute, University of Minnesota Medical School, Minneapolis, MN 55455, USA; shett098@umn.edu; 4 Department of Biological Sciences, University of Minnesota Medical School, Minneapolis, MN 55108, USA

**Keywords:** stroke, macrophage, microglia, neurogenesis, neuroprotection, inflammation, transplantation, therapies

## Abstract

Stroke remains the number one cause of morbidity in the United States. Within weeks to months after an ischemic event, there is a resolution of inflammation and evidence of neurogenesis; however, years following a stroke, there is evidence of chronic inflammation in the central nervous system, possibly by the persistence of an autoimmune response to brain antigens as a result of ischemia. The mechanisms underlying the involvement of macrophage and microglial activation after stroke are widely acknowledged as having a role in ischemic stroke pathology; thus, modulating inflammation and neurological recovery is a hopeful strategy for treating the long-term outcomes after ischemic injury. Current treatments fail to provide neuroprotective or neurorestorative benefits after stroke; therefore, to ameliorate brain injury-induced deficits, therapies must alter both the initial response to injury and the subsequent inflammatory process. This review will address differences in macrophage and microglia nomenclature and summarize recent work in elucidating the mechanisms of macrophage and microglial participation in antigen presentation, neuroprotection, angiogenesis, neurogenesis, synaptic remodeling, and immune modulating strategies for treating the long-term outcomes after ischemic injury.

## 1. Introduction

In the United States, stroke is the number one cause of morbidity and the third highest cause of death. It is estimated that over 800,000 people have a stroke each year, equating more than one stroke-per-minute. Of these 800,000 strokes, 87% are ischemic in nature, resulting from a clot or mass blocking a blood vessel, and 13% are hemorrhagic caused by a weakened blood vessel that ruptures [1]. Immediately after a stroke, damage-associated molecular patterns (DAMPs) activate microglia. At the injury site, cytokines and chemokines produced by activated microglia, endothelial cells, and astrocytes allow for transendothelial migration of monocytes and macrophages through the compromised blood–brain barrier (BBB) [2]. The ensuing neuroinflammation causes motor and cognitive deficits and are linked to increased risk of developing neurodegenerative disorders and chronic encephalopathy. 

Despite decades of research, tissue plasminogen activator (tPA) remains the only drug approved by the U.S. Food and Drug Administration for treating stroke. Effective only if administered 3–4.5 h after the onset of stroke, the vast majority of patients are not able to receive tPA [3]. Advances in endovascular approaches such as mechanical thrombectomy have shown to be effective at reducing post-stroke disability in patients when treated within the first 8 h of symptom onset, but only a small number of patients are eligible to receive this treatment [4]. There is a need for therapies that offer neuroprotective and neurorestorative benefits within the limitation of narrow treatment windows. 

Current treatments fail to provide neuroprotective or neurorestorative benefits; therefore, to ameliorate brain injury-induced deficits, therapies must target both the initial response to injury and the subsequent inflammatory process [5]. Recent data from experimental and clinical stroke studies have further elucidated the complex pathophysiology of ischemic stroke. The mechanisms underlying the involvement of microglia and macrophages in both neuroprotection and neurogenesis after stroke are widely acknowledged as having a role in ischemic stroke pathology [6,7,8,9] Since microglia and macrophages are regarded as major players in the pathological progression of ischemic stroke, modulating inflammation and neurological recovery is a hopeful strategy for treating the long-term outcomes after ischemic injury. 

## 2. Nomenclature

### 2.1. Microglial Classification

Microglia are the resident macrophages in the central nervous system (CNS) and are a part of the mononuclear phagocyte system. Similar to other tissue-specific macrophages of embryonic origin, microglia originate from the yolk sack and are established approximately at the same time as neurons during the early prenatal period, where they participate in the developmental maintenance of neurons throughout life [10]. However, unlike peripheral macrophages, microglia are the only adult population with an early erythroid myeloid progenitor origin, which originate in the yolk sac [11]. Due to the maturation of the BBB, the CNS does not receive additional precursors from postnatal hematopoiesis; therefore, microglia remain yolk sac-derived myeloid cells exclusively in the CNS [12,13,14]. 

### 2.2. Macrophage Classification

Macrophages that reside in the CNS-border, which includes the choroid plexus, perivascular spaces, and subdural meninges, express distinct transcriptional signatures from microglia and infiltrating macrophages [15,16]. Through fluorescence-activated cell sorting, other microglia-specific genes have been identified as Tmem119, P2ry12, and P2ry13 [17], while systemic macrophage-specific genes include CD163 and Fabp4. Under physiological conditions, macrophages, except microglia and monocytes, express CD163 [18]. Different inflammatory signals will either upregulate (anti-inflammatory) or downregulate (pro-inflammatory) CD163 expression [19], in which CD163+ cells proliferate in the injured brain 3–4 days following ischemia [16]. Various gating strategies can be used to further separate macrophages at CNS interfaces from microglia [15]. 

### 2.3. Discrepancies in Nomenclature

Although current advances in technology have defined microglia and macrophages as having transcriptionally distinct profiles, identification of microglia and macrophages in the brain through the use of various cellular markers remains unclear and contentious in the literature (Table 1). Many groups consider all macrophages in the brain as microglia and utilize ionized calcium binding adaptor molecule 1 (Iba1)+ staining as evidence, but Iba1 can label both resident microglia and macrophages. Similarly, many other macrophage populations including microglia and CNS-associated myeloid cells express CD11b, CD115, Iba1, and F4/80 [20,21]. Due to the historical discrepancies in nomenclature and inability to distinguish between microglia and macrophages, many groups refer to CNS mononuclear phagocytes and recruited peripheral blood monocytes collectively as “microglia/macrophages.” More recently, cellular profiling can also be determined through single cell RNA-Seq and flow cytometry, whereas CD11b+CD45^high^ populations are identified as macrophages and CD11b+CD45^low^ populations as microglia. However, it is important to note that microglia can upregulate CD45 after ischemic stroke and become indistinguishable from CD11b+CD45^high^ cells in the border regions of the CNS or the periphery [21,22]. 

In this review, we will distinctly denote microglia and macrophage nomenclature based on the methods by which each research study used to make its conclusions, with additional comments on the authors’ chosen nomenclature and any potential discrepancies with the study’s interpretation resulting from that choice.

## 3. Dichotomous Role of Microglia and Macrophages

### 3.1. Microglial Activation during Stroke

**Figure 1:**
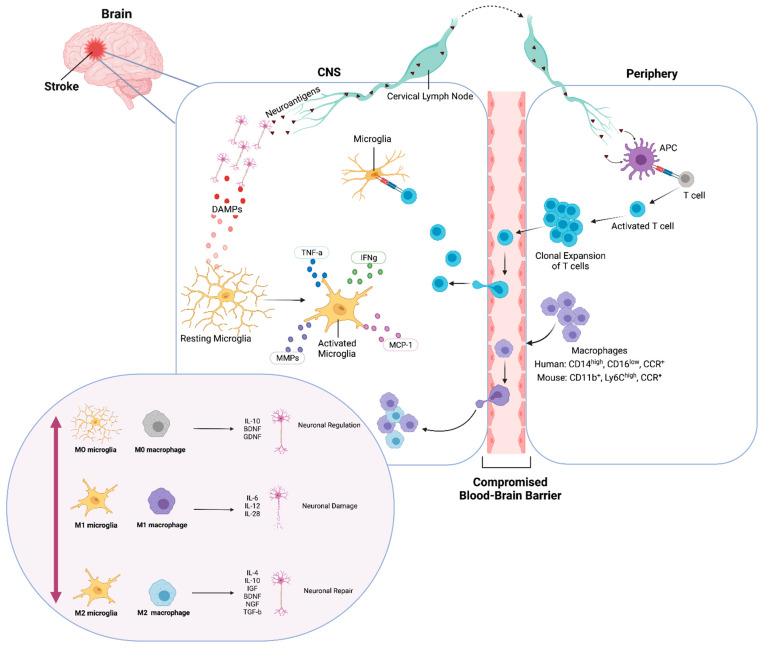
Ischemic inflammatory response of microglia and macrophages. Immediately after an ischemic event, DAMPs activate resting (M0) microglia and neuroantigens are released. Microglia produce cytokines and chemokines. Transendothelial migration of monocytes and macrophages occurs through the compromised BBB. Neuroantigens are processed and presented by APCs and activate CD4+ T-cells, which undergo clonal expansion, promoting inflammation and neuronal damage. Classically activated (M1) microglia and macrophages release pro-inflammatory factors and contribute to neuronal damage. Conversely, alternatively activated (M2) microglia and macrophages release anti-inflammatory factors and contribute to neuronal repair and neurogenesis. Created with BioRender.com.

After the onset of brain ischemia, microglia undergo morphological and functional changes in the penumbra, while macrophages also infiltrate the brain parenchyma and migrate toward the infarct area. Studies have shown that activated microglia can have both a beneficial and detrimental effect during all stages of ischemic stroke; however, the timing and kinetics at which specific anti- and proinflammatory events occur may influence the nature of the outcome (Figure 1). Activated microglia release cytotoxic factors that can exacerbate ischemia and enable poststroke inflammation, displaying elevated levels of reactive oxygen species and TNF [43]. Several reports maintain this detrimental role of microglia in stroke; however, these reports are not consistent with their markers, often looking broadly at Iba1+ or CD11b+ cells, and sometimes using glial fibrillary acidic protein (GFAP), a marker often used to label reactive astrocytes to identify microglia.

While the detrimental effects of microglia have been emphasized in many studies, Szalay et al. showed that a selective elimination of microglia using a CSF1R antagonist PLX3397 prior to a middle cerebral artery occlusion (MCAO) led to a 60% increase in infarct size. The increase in infarct volume was reversed with microglial repopulation [43]. Similarly, Herbert et al. reported a neuroprotective role of proliferating microglia in stroke after selectively ablating proliferating microglia using the Galectin-3 (Mac-2) marker to preferentially label resident microglia [44]. The result was an altered proinflammatory brain response and exacerbation of ischemic injury. Notably, these studies abrogated the microglial response to stroke early in the injury timeline, resulting in detrimental effects.

### 3.2. Macrophage Activation Profiles

There has been evidence of infiltration of peripheral monocytes to the brain in response to DAMPs produced after ischemic stroke, with the presence of neutrophils and macrophages in the site of CNS injury [45,46,47,48] (Figure 1). Activated macrophages can be categorized on a spectrum of functional activity, starting at resting (M0), classical activation (M1), which promotes inflammatory responses, to alternate activation (M2), which promotes tissue remodeling, wound healing, and immune regulation [49,50]. 

This switch in macrophage phenotype has been explored in ischemic models. For example, monocyte chemoattractant protein-1 (MCP-1) possesses cytokine-like properties and plays a significant role in macrophage and Ly-6C^hi^ (CCR2+) proinflammatory monocyte migration to injury sites. The increase in CCL2 (MCP-1) expression in the ischemic hemisphere leads to an increased infarct volume [51,52], while the inhibition of CCR2 or MCP-1 resulted in a reduced infarct size [53,54]. In contrast, Ly-6C^lo^ monocytes that do not express CCR2, but express the CX3CR1 receptor for CXC3CL1 fractalkine, develop into M2 macrophages after recruitment to normal tissues [55]. This understanding has led to many studies to focus on skewing microglia and macrophage toward the M2 phenotype rather than M1. 

It is important to note that the M0/M1/M2 trichotomy is an oversimplification of activation states as the status of macrophages may include overlapping functional phenotype as well as different ones. For example, the M2 phenotype includes subpopulations such as M2a, M2b, M2c, and Mox, each with distinct physiological functions [56]. Not all current studies in CNS injuries have characterized these subpopulations, so while the M0/M1/M2 classification is broad, it is still a meaningful concept to facilitate our understanding of the functional status of macrophages.

## 4. Neuroprotection after Stroke

Depending on the ischemic milieu, macrophages can transform to different functional phenotypes in the hemisphere of infarction [57]. In response to signals from the microenvironment, microglia and recruited macrophages are alternatively activated and express an M2 phenotype during the early stages of ischemic stroke and then transform over time into an M1 phenotype in the infarct lesion [58]. Others have suggested the opposite effect, in which Ly6C^hi^ (CCR2+) macrophages express the M1 phenotype in the ischemic hemisphere, but convert to an alternate phenotype at the injury site 48 h after stroke. These proinflammatory cells downregulate Ly6C and CCR2 expression, enhancing the resolution of inflammation by releasing vascular endothelial growth factor and TGF-β [59,60]. Zhang et al. reported the reprogramming of infiltrating macrophages that strongly favored efferocytic activity 3–7 days after brain ischemia and were potentially regulated by PPARy and STAT6 [61]. Similarly, Wang et al. also reported RNA transcriptome profiles that were related to the regulation of cell migration and mobilization and pro-neurovascular remodeling effects in macrophages as early as five days following ischemic stroke [21]. 

### 4.1. Angiogenesis

Microglia are known to influence angiogenesis, whereas the elimination of microglia or deficiency in macrophage colony-stimulating factory causes a reduction in retinal vasculature [62,63]. During ischemic stroke, Iba1+ microglia and macrophages cluster around the vasculature and release vascular endothelial growth factor to promote the reconstruction of blood vessels following stroke [64,65,66]. Pro-angiogenic microglia and macrophages may enhance neural proliferation and differentiation following stroke, possibly contributing to neurogenesis and CNS repair. 

### 4.2. Synaptic Remodeling

It is often reported that ischemic stroke can lead to synaptic dysfunction; however, it is known that microglia and macrophages participate in synaptic remodeling and refining neural circuitry [67]. Microglia promote spine formation and synaptic maturation through CX3CR1 and complement proteins as well as synaptic pruning in the brain during development [68,69,70]. Modulation of synaptic function and the neural circuitry is also dependent on the activation state of microglia and macrophages. For example, one of the inflammatory mediators of neurotoxicity in stroke is NADPH oxidase, which is triggered by CR3 activation on macrophages and microglia, resulting in long-term synaptic depression [71,72]. Modulation of activation states of both microglia and macrophages may thus change the inflammatory environment following ischemic injury, encouraging neuroprotection and angiogenesis. 

## 5. Neurogenesis after Stroke

Macrophage and microglial activation have been linked to changes in neurogenesis following ischemic stroke in mammals [9,73]. It has been suggested that newly generated neuroblasts from the subgranular zone (SGZ) of the hippocampus and the subventricular zone (SVZ) of the lateral ventricles can migrate and terminally differentiate into specific cell types in order to generate new granule cell neurons in the hippocampal dentate gyrus as anatomical substrates for learning and memory [74], or replenish the loss of neurons in other regions of the adult brain [75,76], respectively. Some studies suggest that the brain is capable of self-repair after insults of extensive neuronal death through a number of compensatory neurogenesis mechanisms after stroke [77,78,79]. Additionally, enhanced SVZ neurogenesis has been observed after stroke [80,81], and newborn neurons have been found in the ischemic penumbra [82,83]. 

Microglia and macrophages may participate in regulating neurogenesis by supporting axonal regrowth and regeneration to allow for functional recovery after stroke. Their production of local trophic gradients helps to stimulate axonal sprouting toward the infarct area [84]. Microglia also are necessary for regulating synaptic maturation, while in the setting of microglial injury, there can be synaptic dysfunction [85]. This active regulation of functional synapses in the CNS through axon guidance, synaptic patterning, and cell migration is evidence of their role in modulating neurogenesis [86,87,88]. 

The increase in neurogenesis after stroke may only be transient, and similar to what is seen in traumatic brain injury models, appears to be disrupted by a secondary inflammatory response [89]. While the production of trophic factors is essential for the migration of newborn neurons [90], activation of microglia and macrophages can also mediate inflammation that is detrimental to neurogenesis [7,91]. CD4+ T cells are essential to maintain homeostatic neurogenesis [92], and they also contribute to learning and memory [93]; however, activated cells responding to the injury may inhibit neurogenesis [94]. New treatment modalities can arise from shedding light on the mechanisms that modulate neurogenesis after stroke, as currently there are none.

## 6. Therapeutic Perspectives

Although neurons in the stroke lesion core cannot be rescued, improvement in stroke outcome can be achieved through reduction in secondary brain injury due to inflammation. Based on our developing knowledge of microglia and macrophage involvement in the pathophysiological processes of stroke, many novel therapeutic approaches have emerged to promote remodeling of the injured brain in stroke models by altering the activation phenotypes of these cells. 

### 6.1. Polarization via Small Molecules

**Figure 2:**
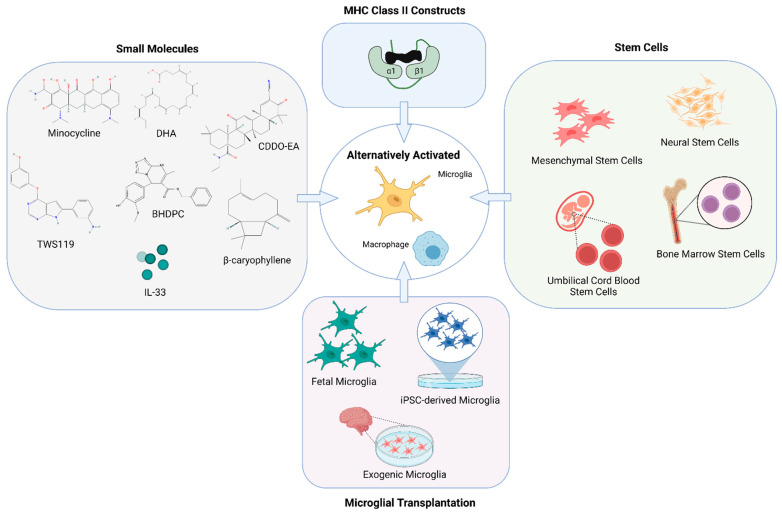
Modulating microglia and macrophage for therapy. Many novel therapeutic approaches have emerged to promote remodeling of the injured brain in stroke models by altering the activation phenotype of microglia and macrophages. Methods for alternative activation of microglia and macrophages include but are not limited to the use of various small molecules, partial MHC class II constructs, stem cell therapies as well as transplantation of microglia as a treatment for both the acute and chronic effects of stroke. Created with BioRender.com.

Modulating microglial and macrophage activation and polarization through use of various pharmacological and small molecules has been a popular area of study (Figure 2). Minocycline, a tetracycline antibiotic commonly used as an inhibitor of microglial activation, administered five times a week in amyotrophic lateral sclerosis (ALS) mice from 8–24 weeks of age, diminished the expression of M1 microglia and macrophages but not M2 microglia and macrophages [95]. Additionally, treatment with minocycline for one week, beginning at four days after reperfusion injury in rats, preserved adult new neurons, reduced reactive astrocytes, and improved dentate gyrus neurogenesis and neurological function [96]. Typically, ischemic stroke is characterized by the downregulation of the Wnt/beta-catenin signaling pathway; however, activation of Wnt/beta-catenin signaling through TWS119 attenuated neuroinflammation after stroke, by driving microglial anti-inflammatory activation, promotes angiogenesis [97,98]. 

The nature of the ischemic environment is a key determinant in driving microglia and macrophage function and their activation phenotype. For example, microglia and macrophages are highly susceptible to energy deficits, leading to activation and recruitment; thus, metabolic status of the lesion environment is a major factor in determining the nature of microglial response [99,100]. A number of studies have examined the effects of small molecules on the shift in microglial polarization toward an M2 phenotype after an ischemic event. One study found that a pyrimidine derivative, BHDPC, could confer neuroprotective actions and suppress both microglial and macrophage activation by inactivating NF-κB signaling, but promoted M2 polarization through BHDPC-enhanced phosphorylation of protein kinase A and cAMP-response element-bind protein in rats [101]. 

Microglia and macrophage activation could also be modulated toward the M2 phenotype through post-stroke treatment with docosahexaenoic acid (DHA) administered immediately after reperfusion and daily for three days thereafter. Treatment with DHA also significantly inhibits infiltration of neutrophils, T, and B lymphocytes [102]. A polarization shift of microglia and macrophages toward the M2 phenotype with a reduction in neurological deficit and infarct volume was also seen when a redox transcription factor NFE2 related factor 2 activator, CDDO-EA, was administered 30 min after the end of the ischemic period in mice [103]. Modulation of microglial M2 polarization via the toll-like receptor 4 pathway, a regulator of macrophage activation and polarization after injury, through the use of β-caryophyllene, a natural bicyclic sesquiterpene, also reduced infarct volume and neurologic deficits in mice after a transient MCAO [104]. Additionally, human stroke patients have increased plasma levels of sST2, an inhibitory IL-33 receptor; while in mice, treatment with IL-33, a cytokine known to induce a shift toward M2 polarization, increased peripheral levels of IL-4 in the spleen and peri-infarct area [105]. 

Angiogenesis can also be stimulated by modulating microglial polarization toward M2 through AMPK signaling after stroke. The treatment of human umbilical vein endothelial cells (HUVECs) in vitro with conditioned media collected from BV-2 microglial cells in culture incubated in berberine, an isoquinoline alkaloid extract from traditional Chinese medicine, facilitated angiogenesis of HUVEC cells [106]. Moreover, oral administration of berberine in mice subjected to transient MCAO resulted in angiogenesis as revealed by PET/CT imaging.

Additionally, the use of partial major histocompatibility complex (MHC) class II constructs as a novel treatment for ischemic stroke presents hopeful outcomes (Figure 2). These constructs have demonstrated a reduction in infarct volume and long-term neurological deficit in both young and aging mice ischemic models [107]. The mechanism of action of partial MHC class II constructs involves inhibiting the infiltration of immune cells into the CNS and reversing stroke-associated splenic atrophy, thereby also providing a therapeutic effect in the periphery [108]. Partial MHC class II constructs have been shown to enhance alternatively activated M2 microglia and macrophages [109].

It is also worth mentioning that there is accruing evidence suggesting oxidative stress activated mechanisms playing a role in the impairment of function after stroke. It is known that M1 microglia produce free radicals and oxidants, leading to long-term deficits in stroke patients [110,111,112,113,114]. When oxidative enzyme myeloperoxidase (MPO) activity was inhibited after stroke, there was a noticeable reduction in the number of M1 microglial cells, but there was no impact on M2 microglia. Additionally, there was an increase in proliferation and differentiation of neural stem cells and protection of exogenous neural cells [111,115]. Modulating microglial phenotypes by mediating oxidative stress and neuroinflammation can affect adult neurogenesis in the post-stroke brain.

### 6.2. Stem Cell Therapies

Stem cell therapies for stroke have the potential to provide neurorestorative benefits. The type of stem cell and route of administration determines the efficacy of stem cell-based therapies in mediating a therapeutic effect (Figure 2). The therapeutic of effect of neural stem cells have been attributed to the promotion of neurogenesis and regeneration.

Neural stem cell (NSC) transplants have been shown to improve behavioral outcomes and angiogenesis in rat models of ischemic stroke, leading to studies interrogating human NSCs (hNSC) [116,117]. Grafted hNSCs can differentiate into neurons, astrocytes, and oligodendrocytes in the ischemic brain, even making direct contact with stroke-damaged vasculature and participating in remyelination, respectively [118]. More recently, administration of clinical-grade hNSC line CTX0E03 by intracerebral implantation has been shown to improve upper limb function in stroke patients [119]. Success in previous clinical trials will allow researchers to utilize this cell line for other neurological diseases and disorders.

Bone marrow stem cells (BMSC) have been shown to migrate to the site of ischemia and differentiate into neural cells [120]. More recently, bone marrow-derived mesenchymal stem cells (MSC) SB632 were associated with improvement in clinical outcome in stroke patients with chronic motor deficits [121]. These cells were intracerebrally transplanted and well tolerated after 12 months. The advantages of MSC transplantation for functional recovery, angiogenesis, and endogenous neurogenesis have also been shown in animal stroke models [122,123,124,125]. However, one major limitation of the current stem cell therapies is the sparse migration of MCSs to the ischemic brain regions after transplantation.

Umbilical cord blood stem cells (UCBSC) offer many advantages over other types of stem cells. The immune properties of UCBSCs allow for increased tolerance for human leukocyte antigen mismatches and decreased incidence of graft-versus-host disease as well as ease of procurement and availability [126,127]. Non-hematopoietic umbilical cord blood stem cell (nh-UCBSC) has also been shown to ameliorate ischemic brain injury by reducing the number of macrophages and microglia and normalizing the number of B cells and T cells in the brain following stroke when administered 48 h after the ischemic injury [128,129]. The major alterations in gene expression profiles after the administration of nh-UCBSCs has the potential for altering microglia and macrophage activation to improve macrophage-induced neural damage.

## 7. Chronic Stroke

A significant subset of stroke patients experience progressive cognitive decline. Levine et al. conducted a prospective study of cognitive function in 23,572 patients aged 46 years or older who were followed for an average of six years post stroke [130]. These investigators reported a decline in global cognition, new learning, and verbal learning during the acute phase following stroke. During the subsequent years of monitoring, they found that individuals with stroke exhibited faster rates of decline, global cognition, and executive function when compared to individuals without stroke. Pendlebury and Rothwell published a meta-analysis of 7511 subjects in 73 reports of stroke and dementia [131]. Of these stroke subjects, 10% developed dementia after their initial stroke, while approximately 33% developed dementia after a recurrent stroke. Contributions to cognitive decline may stem from the interaction of immune cells, the presence of neuroantigens, and sustained microglial activation, resulting in chronic inflammation.

### Antigen Presentation and Cognitive Decline

After an ischemic event, antigen-presenting cells (APCs) accumulate in the brain parenchyma and express MHC class I and II cell surface molecules [132,133,134,135]. APCs engulf and process the peptides of damaged and dying cells and then present these peptides to CD8+ T cells, in association with MHC class I, and to CD4 T+ cells, in association with MHC class II (Figure 1). The activation of the T cell population to target self-antigens in the CNS can potentially contribute to the progressive, cognitive decline seen in many patients after stroke. Macrophage populations of dendritic cells that express both Iba1 and CD11lb markers migrate in the choroid plexus in mice and behave as APCs [136,137]. A population of Iba1+CD11b+CD45^int^ are a subset of microglial cells that can also upregulate antigen presentation and activation markers [138,139,140]. While CD4+ T-cell mediated neuroprotection is initiated by APCs, MHC class II+ microglia APCs are required for a secondary restimulation and can drive antigen-specific neuroprotection [141], demonstrating a close relationship between these two cell types. For example, in the brain of Alzheimer’s disease (AD) patients, the transition into AD dementia correlates with increased MHC class II+ microglia-mediated immunity and a decrease in T cell number [141,142,143].

The volume of infarction and severity of stroke is correlated with the concentration of neural antigens found in the serum of patients after stroke [144,145]. Autoantibodies against brain antigens have been found in the CSF of patients with stroke, possibly contributing to the development of post-stroke cognitive impairment [146,147,148]. A series of studies demonstrated the reversal of stroke-associated splenic atrophy 96 h after MCAO by administering partial MHC class II constructs starting at 4 h after the onset of ischemia [107,109,149,150,151]. These MHC class II constructs inhibited neuroantigen-specific T-cells and blocked the binding of macrophage migration inhibitory factor to its CD74 receptor, promoting M2 macrophage and microglia phenotypes in the CNS and also improving long-term cognitive outcomes 28 days after stroke [109,151]. Thus, the balance between macrophage and microglia phenotypes and presence of neuroantigens following a stroke is critical to the outcome of ischemic brain injury.

## 8. Microglial Transplantation as a Treatment for Chronic Stroke

Regulation of microglial function as a means to attenuate neurological dysfunction seen in chronic stroke has become a promising strategy in pre-clinical research [152]. Cell transplantation therapies involving microglia have been shown to exert a therapeutic effect for Alzheimer’s disease [153]. While other transplanted cells types such as mesenchymal stem cells are more common [154], the therapeutic potential of microglia transplantation in treating chronic stroke is an emerging field (Figure 2).

Positron emission tomography imaging studies in patients with chronic stroke and cognitive decline revealed persistent microglia activation [155]. Preclinical animal models of chronic stroke support the contention that chronic inflammation and sustained microglia activation may be an underlying factor in the progressive deterioration observed in the clinical setting. Basu et al. demonstrated that IL-1 and IL-1R signaling is essential for progressive neurodegeneration that arises subsequent to ischemic brain injury [156]. In addition, Yang et al. reported that the ST2 receptor, a member of the IL-1R receptor family, is critical for microglial signaling related to ischemic brain injury [157]. Together, these results suggest that targeting microglial signaling pathways or replacing chronically active inflammatory microglia may offer a therapeutic strategy for ameliorating progressive neurodegeneration and cognitive decline resulting from ischemic stroke.

### 8.1. Transplantation of Fetal Microglia

Transplantation of fetal microglia has been demonstrated to improve ischemia-induced functional changes and apoptotic events after stroke. Ischemia was induced in rats by MCAO and human microglial cells (HMO6) from fetal telencephalon tissue were transplanted into a treated group [158]. Animals that received HMO6 transplantation showed significantly reduced infarct volume and apoptotic cells in the infarct core and penumbra when compared to the control group that did not receive the transplant. Gene expression analysis showed that HMO6 cells migrated to the ischemic area and produced neurotrophic factors such as GDNF and BDNF and anti-inflammatory cytokines IL-4 and IL-5, which reduced the endogenous glial response. The accumulation of transplanted microglia in the lesion core suggests that microglia can potentially be used in gene therapy as a vehicle for the transfer of therapeutic genes.

Fetal microglial transplantation may improve stroke outcomes by modulating inflammation and facilitating angiogenesis. Expression of IL-1β is high in HMO6 and has been shown to increase VEGF mRNA expression in HMO6 cell lines when co-cultured with a mesenchymal stem cell line B10 [159]. White matter lesions (WMLs) as a result of chronic cerebral ischemia are thought to contribute to vascular dementia. HMO6 cells were injected intravenously and WML development was assessed in a chronic cerebral hypoperfusion rat model induced by bilateral common carotid artery occlusion (BCAO) [160]. The authors found that the transplantation of HMO6 inhibited BCAO-induced WMLs and displayed an early and prolonged improvement in WMLs. Both glial activation and astrocyte and microglial accumulation was inhibited in the site of BCAO after HMO6 transplantation, and this effect was more robust compared to animals that received B10 transplantation. Additionally, the expression of microglial proteases MMP-2, MMP-9, and cathepsin B, all of which influence WML pathology, was also inhibited after transplantation. The capacity of transplanted fetal microglial cells to reduce the severity of WMLs through glial activation should be considered as a potential therapy.

### 8.2. Transplantation of iPSC Derived Microglia

Microglia derived from iPSCs are an important consideration for translational neuroimmunology research and hold great potential for therapy. iPSC-microglia are able to recapitulate the inflammatory-modulating properties of brain-resident microglia by resembling the in vivo phenotypical profiles of microglia within stroke lesions and responding to IL-13 stimulation [161]. While primary microglia grown in culture tend to have major differences in morphology and gene expression when compared to resident-microglia in the brain, single-cell RNA-sequencing of transplanted iPSC-derived microglia showed similarities to healthy primary microglia in both gene expression profiles and phenotypic morphology and are able to functionally integrate in the chimeric mouse brain [162,163]. Beyond iPSC-derived microglia serving as a suitable representation of human microglial cells, the cells have also demonstrated the ability to mitigate neuronal loss after stroke in rats aged 24 months. Animals that received iPSCs also performed better at the cylinder test at four and seven weeks after the ischemic event when compared to animals injected with only the vehicle, suggesting the ability of iPSCs to ameliorate functional deficits in a stroke-injured aged brain [164]. Additionally, it has been shown that IPSC-derived microglia can be transplanted into the brain through a transnasal route, offering a noninvasive method to deliver potential therapies [165].

### 8.3. Prospects for Transplanting Exogenic Microglia

Exogenous microglia also have the potential to protect against neuronal damage after stroke by exhibiting an affinity for ischemic brain lesions. Injection of exogenous microglia was show to promote CA1 cell survival by migrating to the CA1 cell layer and increasing the expression of BDNF and GDNF in the ischemic hippocampus [166]. Cell therapies involving hypoxic preconditioning is becoming a popular strategy for treating ischemic stroke. Microglia subjected to oxygen-glucose deprivation before transplantation can induce anti-inflammatory microglia and result in the overexpression of remodeling factors such as MMP-9, VEGF, and TGF-β in the injured brain parenchyma [167]. Such therapeutic potential has led to hypoxic preconditioning of stem cells to facilitate the switching of microglia toward an anti-inflammatory polarization for use in alleviating ischemic injury [168].

## 9. Conclusions and Perspectives

Ischemic injury is characterized by the time dependent activation of microglia and infiltration of macrophages into the penumbral tissue. Lack of effective treatment options is partially attributed to the dynamic pathophysiology of ischemic stroke; however, future research aimed at elucidating mechanisms underlying the involvement of these cell types in a time-dependent manner is a promising strategy for treating the long-term outcomes after ischemic injury.

The studies reviewed here suggest the importance of microglia and macrophages in both neuroprotection and neurogenesis after stroke and the potential use of these cell types for therapy, especially for treating long-term cognitive deficits. Further discrimination of this relationship may include careful distinction between different populations of macrophage and microglia due to their temporal differences after the onset of stroke. Currently in the literature, there is a lack of consistent agreement in nomenclature used to distinguish differences between microglia and macrophage populations. This aspect will be important to accurately describe the role of these cells over time, shortly before and following the induction of stroke.

While alternative activation and modulation of the macrophage and microglial response may stimulate neurogenesis, angiogenesis, and synaptic remodeling in the injured brain, it is worth considering other immune cells, factors, and molecules that are influenced by inflammatory microglia and macrophages. Investigation of functional recovery and improvement of long-term cognitive outcomes may be dependent on the relationship between macrophage and microglia with MHC class II molecules, T cells, and the presence of neuroantigens.

Understanding the timing and kinetics at which specific anti- and proinflammatory events occur will affect stroke outcome, and delineating the role of macrophage and microglia and their signaling pathways over time could provide a host of candidates for therapeutic interventions after an ischemic event. Transplantation studies demonstrate the potential of targeting signaling pathways and using microglia in gene therapy as a vehicle for the transfer of therapeutic genes as a promising therapy for treating chronic stroke.

The role of microglia and macrophages in stroke-induced neurogenesis is multifaceted and complex: production of trophic factors is essential for the migration of newborn neurons; however, activation of microglia and macrophages can mediate inflammation that is detrimental to neurogenesis. Their interaction with other immune cells such as lymphocytes contributes to the complexity of the microenvironment generated by DAMPs and neuroantigens, and consequently alters the macrophage and microglial response to injury (Figure 1). Since there is a dichotomy in the consequences of microglia- and macrophage-induced neurogenesis and involvement in inflammation through its different activation phenotypes, future research aimed at elucidating the temporal characteristics of activated microglia and macrophages to determine their beneficial role in stimulating neuroprotection and neurogenesis after stroke would be critical for developing novel therapeutic strategies. Strategies targeted toward modulating microglia and macrophage activation of inflammatory pathways have the potential to be advantageous in controlling injury and improving clinical outcomes following stroke.

## Figures and Tables

**Table 1:** Microglia and macrophage markers. Many studies utilize a variety of microglia and macrophage markers. Some of these markers may overlap between the two cell types.

Cell Type	Marker	References
Microglia	C1qa	[23,24]
	CD11b+CD45^low^	[21,22]
	CD11b+CD45^int^	[21,22]
	Fcrls	[24,25]
	Hexb	[26,27]
	P2ry12	[17,24]
	P2ry13	[17,24]
	Tmem119	[17,28]
Microglia and Macrophage	CD115	[20,21]
	CD11b	[20,21]
	CD45	[29,30,31]
	CD68	[30,31]
	Cx3cr1	[24,32]
	F4/80	[21,33]
	Iba1	[34,35]
Macrophage	CD163	[16,18,19]
	CD169	[36,37]
	CD11b+CD45^high^	[21,38]
	CD206^high^	[39,40]
	Fabp4	[41,42]

## Data Availability

Not applicable.
